# TNFRSF11B activates Wnt/β-catenin signaling and promotes gastric cancer progression

**DOI:** 10.7150/ijbs.43630

**Published:** 2020-04-25

**Authors:** Fengming Luan, Xiaomei Li, Xiaojing Cheng, Longtao Huangfu, Jing Han, Ting Guo, Hong Du, Xianzi Wen, Jiafu Ji

**Affiliations:** 1 Key laboratory of Carcinogenesis and Translational Research (Ministry of education), Division of gastrointestinal Cancer Translational Research laboratory, Peking University Cancer Hospital & Institute, Beijing, China;; 2 Department of gastrointestinal surgery, Peking University Cancer Hospital & Institute, Beijing, China.

**Keywords:** gastric cancer, TNFRSF11B, tumorigenesis, Wnt/β-catenin, apoptosis, TRAIL

## Abstract

Tumor necrosis factor receptor superfamily member 11B (TNFRSF11B) has been studied to be involved in the development and progression of several human malignancies. However, little is unveiled regarding the complex mechanisms of TNFRSF11B in human gastric cancer (GC). The clinical significance of TNFRSF11B was assessed in 70 and 160 GC tissues using immunohistochemistry method and gene microarray analysis, respectively. The biological function of TNFRSF11B was studied in vitro and in vivo assays. Immunofluorescence assay was used to evaluate the expression of β-catenin in the nucleus. The expression of β-catenin and related protein was determined by Western blot. The interaction between TNFRSF11B and GSK3β was detected by co-immunoprecipitation. We demonstrated that TNFRSF11B was highly expressed in the cytoplasm of GC and associated with the patient poor outcome. Our studies showed that TNFRSF11B in GC cells significantly promoted cell proliferation, migration, invasion *in vitro* and tumorigenic ability *in vitro* and *in vivo*. Meanwhile, TNFRSF11B inhibited GC cell apoptosis. The proportion of nuclear active β-catenin showed positively correlation with TNFRSF11B expression. TNFRSF11B directly combined with GSK-3β upregulating its phosphorylation, and increased expression of β-catenin and its downstream effectors. Collectively, these findings demonstrate that TNFRSF11B promote the aggressive phenotypes of GC cells and activated Wnt/β-catenin signaling. Accordingly, TNFRSF11B had potential as a biomarker and inhibition of TNFRSF11B expression might offer a new therapeutic target for GC patients.

## Introduction

Gastric cancer (GC) is the fifth most common type of cancer in the world and the third dominating cause of cancer-related deaths 1.The early stages of gastric cancer are usually asymptomatic or just nonspecific symptoms. The cancer was often advanced and had a poor prognosis by the time of diagnosis 2. Adenocarcinoma is the major type of GC. It can be further subdivided into intestinal and diffuse types according to Lauren's classification 3. Although surgical skills for the treatment of GC have been improved and numerous novel chemotherapy regimens have also been developed, the survival rate still keeps low 4.One of the important reasons for the poor prognosis in GC is the invalidity of anticancer agents to target tumor cells and tissues selectively 5.Therefore, it is of great significance to find a promising therapeutic target and a new biomarker to predict the prognosis of gastric cancer.

TNFRSF11B is also termed Osteoprotegerin (OPG). It is a member of the tumor necrosis factor receptor super family (TNFRSF). It is a secreted protein and a soluble decoy receptor for tumor necrosis factor (TNF)-related apoptosis inducing ligand (TRAIL). TNFRSF11B was initially found to bind to the nuclear factor-kappa B(NF-kB) receptor activator, thereby promoting the homeostasis of bone metabolism 6.

It is expressed as a circulating glycoprotein of 401 amino acids with seven structural domains 7 and has been well-characterized as a regulator of bone remodeling via preventing the breakdown of bone and blocking osteoclast maturation 8.The TNFRSF11B gene is located on chromosome 8 at the 8q24 position, which seems to harbor a gene cluster involved in the regulation of bone development and remodeling. Circulating TNFRSF11B can be found either as a free monomer of 60 kDa or as a disulphide bond-linked homodimer form of 120 kDa, which usually has more biologically active than a monomer form 9, 10.

TNFRSF11B has two known TNF family ligands: TNF related apoptosis-inducing ligand (TRAIL) and receptor activator of nuclear factor (NF)-kB ligand 11.In tumor, TRAIL is produced by monocytes in response to interferon (IFN)-γ or -α and is the principal mediator of acquired tumor killing activity of these cells. TRAIL binds membrane-bound receptors carrying death domains(DR4 and DR5) [12]and a decoy receptor.TNFRSF11B competes for the binding of TRAIL to death activated receptors, providing a mechanism to prevent apoptosis in the presence of TRAIL.Therefore,TNFRSF11B expression may be an appropriate strategy to avoid TRAIL- induced apoptosis, leading to the development and progression of tumor. In addition, TNFRSF11B also induces angiogenesis, one of the hallmarks of cancer, thus facilitating tumor growth 6.

Recent research has shown that TNFRSF11B is involved in the development and progression of several human malignancies 13-21. In gastric cancer, high expression of TNFRSF11B was found in gastric cancer cell lines, primary cancer and bone metastasis tissues. And its high expression was closely related with deep infiltration, lymph node metastasis and poor prognosis 22, 23. Even its copy number gains were associated with poor survival in GC patients, particularly in noncardia GC patients 24. However, there are no studies for the biological function and molecular mechanism of TNFRSF11B in gastric cancer. In our study, we aimed to clarify the molecular roles of TNFRSF11B in gastric cancer. We suggested that TNFRSF11B is potentially useful as a prognostic biomarker and could be a novel therapeutic target for gastric cancer.

## Materials and Methods

### Bioinformatic analysis of TNFRSF11B expression in cancer and normal tissues

We used the paired-sample *t*-test for multiple testing to identify the expression of TNFRSF11B in gastric adenocarcinoma tissues(n=415) and normal tissues (n=34) from the Cancer Genome Atlas(TCGA) project gastric cancer database and in a range of gastric cancers (gastric adenocarcinoma n=173, diffuse gastric adenocarcinoma n=45,gastric intestinal type adenocarcinoma n=41, gastric papillary adenocarcinoma n=3,gastric tubular adenocarcinoma n=31, mucinous gastric adenocarcinoma n=12, signet ring cell gastric adenocarcinoma n=2) from the Oncomine database.

### Cell culture

Human GC cell lines, including HGC-27, BGC-823, MGC-803and SGC-7901, were purchased from Cell Bank of the Chinese Academy of Sciences. All the GC cells were cultured in DMEM(GIBCO, Carlsbad, NY, USA)with 10% Fetal bovine serum (FBS)(GIBCO, NY, USA) and 1% antibiotics at 37ºC in a humidified incubator under 5% CO2 condition.

### Tissues sample from patients with gastric cancer

The human gastric tumor samples of 70 paraffin- embedded were obtained from Peking University Beijing Cancer Hospital between October 2005 and December 2010. The gastric cancer patients all underwent radical gastrectomy. All patients were followed until 2015. And another total of 160 GC tissues were obtained from patients who were diagnosed and underwent radical resections at Peking University Cancer Hospital between 2007 and 2010 and followed up to 2016. All patients with gastric cancer signed the informed consent, and this was conducted in accordance with the Declaration of Helsinki. The tissues collection was approved by the Ethics Committee of Beijing Cancer Hospital. Clinicopathological data and follow-up information was collected from patient data by us. Gastric cancer staging was based on the 2018 tumor node metastasis (TNM) grade recommended by the American Joint Committee on Cancer (AJCC 8th edition). T and N grades were evaluated according to the final pathological results, and M grades were determined according to the surgical results. Overall survival (OS) was calculated from the date of initial surgery to the date of tumor death or the last follow-up. All of the patients received no preoperative chemotherapy or radiotherapy. This study was approved by the ethics committee of Beijing cancer hospital affiliated to Peking University (Approval name: The research on protein molecular network in gastric cancer, Approval number: 2015KT40).

### Immunohistochemistry

Four micron sections were extracted from formalin fixed 70 paraffin-embedded (FFPE) tissues, dewaxed in xylene, and washed with graded alcohol for rehydration. The antigen retrieval was carried out in 0.01M citrate buffer (pH 6.0) by autoclaving for 3 minutes, and then immersed in methanol with 3% hydrogen peroxide for 10 minutes to block endogenous peroxidase activity. Then the sections were blocked by normal sheep serum (DAKO, Germany) for 1 hours at room temperature and after that incubated with TNFRSF11B polyclonal antibody (Abcam, MA, USA, Cat No: ab73400) diluted at 1:1000 for the night at 4°C. We used diaminobenzidine as a chromogen and hematoxylin was used as redyeing agent. The Samples were considered TNFRSF11B- positive when TNFRSF11B staining was present in the cytoplasm of 10% or more cancer cells. TNFRSF11B expression was independently evaluated by two experienced pathologists who had no knowledge of the patient's clinical outcomes. The two pathologists had a high degree of agreement, with a small number of differential cases (<5%) reaching agreement after joint review.

### RNA extraction and gene microarray analysis

Total RNA was extracted using Trizol reagent (Invitrogen, Carlsbad, CA, USA) according to the manufacturer's instructions. The RNA was quantified by NanoDrop 2000. The 160 samples were used to synthesize double-stranded cDNA, and the cDNA was then labeled and hybridized to the Gene Expression Microarray (CapitalBio platform v 4.0, CapitalBio Technology, China) according to the manufacturer's protocol. After hybridization, the arrays were washed, and the slides were scanned with an Agilent Microarray Scanner. Raw data were extracted as pair files using the Agilent Feature Extraction. The random variance model was used to identify the differentially expressed genes. The paired t-test was used to calculate the P-value.

### TNFRSF11B was stably overexpressed and silenced in GC cells by lentiviral vector transduction

According to the manufacturer' s instructions, Overexpression Lentivirus was produced via the co-transfection of 293T cells with a pEZ-lv105 vector (pEZ-Mock or pEZ-TNFRSF11B) and lentiviral packaging mix (Invitrogen, Carlsbad, CA,USA). According to the manufacturer's instructions, Knockdown Lentivirus was produced by the co-transfection of 293T cells with a piLenti vector (pEZ-Scramble or pEZ-shTNFRSF11B) and lentiviral packaging mix (Invitrogen, Carlsbad, CA, USA). After transfection for 48 hours, Lentivirus-containing supernatant was collected, centrifuged, and stored at -80°C. For transfection of the virus, 1 mL of the Mock or TNFRSF11B lentiviruses was incubated with HGC- 27 and BGC-823 cells overnight at 37°C in a 5% CO_2_ humidified cell culture incubator. Stable GC cells with increased endogenous TNFRSF11B expression were selected via culturing in puromycin (1 μg/ml).1 mL of the Scramble or shTNFRSF11B lentiviruses was incubated with MGC-803, SGC-7901 cells overnight at 37°C in a 5% CO_2_ humidified cell culture incubator. Stable GC cells with decreased endogenous TNFRSF11B expression was selected via culturing in puromycin (1 μg/ml).

### Quantitative real-time PCR (qRT-PCR)

According to the manufacturer's instructions, total RNA was extracted from cultured cells with Trizol reagent (Invitrogen, Carlsbad, CA, USA). cDNA was generated from 1 μg total RNA with a first-strand cDNA synthesis kit (Promega, Madison, WI, USA); 1 μL of cDNA was amplified with SYBR-Green in 20 μL reactions. The following primers were used to detect the expression of TNFRSF11B and GAPDH (internal control):

TNFRSF11B (Forward): 5'-CTGGAACCCCAGAGCGAAAT-3'; TNFRSF11B (Reverse): 5'-GCGTTTACTTTGGTGCCAGG-3'; GAPDH (Forward): 5'-GACCCCTTCATTGACCTCAAC-3'; GAPDH (Reverse): 5'-CTTCTCCATGGTGGTGAAGA-3'.

The protocol was carried out according to the manufacturer's instructions. The results were standardized to the expression of GAPDH.GAPDH was used as an internal control. The experiments were performed in triplicate.

### Western blot analysis

All cells were lysed in RIPA lysis buffer, (Pierce Biotechnology, Rockford, IL) containing a protease inhibitor cocktail (Roche, Basel, Switzerland) for 30 minutes on the ice, and centrifuged at 15,000×g for 20 minutes. Proteins were separated by 10% sodium dodecyl sulfate-polyacrylamide gel electrophoresis (SDS-PAGE) and transferred onto a 0.45 µm polyvinylidene difluoride (PVDF) membrane (Whatman, Germany). The membrane was blocked with 5% skim milk (pH 7.6) for 1 hour at room temperature, and then incubated with primary antibodies diluted overnight at 4°C. The membrane was washed in Tris Buffered Saline Tween (TBST) and incubated with a diluted horseradish peroxidase (HRP)-conjugated secondary antibody (1:5000) for 1 hours at room temperature. Finally, the membrane was developed using a chemiluminescence detection system (Amersham Imager600, GE, USA). The protein levels were normalized to GAPDH. The densities of the proteins were quantified with Image J software.

### Cell proliferation assay

According to the xCELLigene manufacturer's instructions, the baseline was measured and 10% fetal bovine serum was added to the lower chamber. Cells were seeded in a RTCA E-Plate 16 (RTCA, xCELLigence Roche, Penzberg, Germany) at 2×10^3^ cells per well. The plates were incubated at room temperature for 30 minutes before measurements began. The cell index was measured in a time resolved manner (every 10 min during 96 h). The experiments were performed in triplicate.

### Transwell migration and invasion assays

Cell migration was evaluated with wound-healing and transwell assay. For the wound-healing assay, cells (3×10^4^cells/well) were seeded into 96-well culture plates (Corning-Costar, NY, USA). When cells reached >90% confluence, at 12 hours after seeding, wound scratches were made by a 96-pin WoundMaker (Essen BioScience), and relative wound densities were measured by the IncuCyte Zoom system over a 2-day period. The experiments were performed in triplicate.

In transwell chamber migration and invasion assays, 3×10^4^ cells were put into each chamber, providing with serum-free medium, and permitted to pass through a polycarbonate filter, which had been either precoated by 100 μg Matrigel (Becton Dickinson, San Jose, CA) for the invasion assay or left uncoated for the migration assay. The outside of chambers was filled with DMEM, containing 10% FBS. Cells on the upper surface of the filters were erased after 24 hours (SGC-7901) or 48 hours (HGC-27, BGC-823 and MGC-803) in migration assays. Cells on the upper surface of the filters were erased after 48 hours (SGC-7901) or 72 hours (HGC-27, BGC-823 and MGC-803) in invasion assays. The membranes were fixed with methanol for 15 minutes and stained with 0.5% crystal violet for 15 minutes. The cells on the underside of the filter were photographed and counted in five randomly selected microscopic views.

### Colony formation assay

Cells (500cells/well) were seeded in 6-well plates (Corning-Costar, NY, USA). The medium was changed at regular intervals. After a period of cultivation (2 weeks), adherent cells were washed thrice with PBS and fixed with methanol for 15minutes at room temperature. Colonies were dyed with 0.5% crystal violet for 15 minutes and then washed with distilled water and air-dried. The colonies were counted by light microscopy. Experiments were performed in triplicate.

### Flow cytometric analysis

#### Cell cycle assay

Cells were synchronized in the G0/G1 phase via incubating them in a serum-free medium for 24 hours, and then with DMEM and 10% FBS for 24 hours. Then the cells were trypsinized and washed three times with PBS. Afterwards the cells were fixed with 70% ethanol for 24 hours at -20°C.The samples were washed with PBS and dyed with PI/RNase staining buffer (BD Biosciences) for 15 minutes. Cell cycle analysis was performed via using fluorescence flow cytometry on a FACScan machine (BD Biosciences, San Jose,CA, USA).

#### Apoptosis assay

Per well 5×10^5^ cells were cultured on 6-well plates (Corning-Costar, NY, USA) for 24 hours at 37˚C in a 5% CO2 condition. After incubation, the adherent cells were detached with 0.25% trypsin/ 0.01% EDTA. The detached and suspended cells were collected in DMEM with 10% FBS and centrifuged at 1,500 rpm for 5 minutes. Cells were washed with 1× PBS and stained with 200 µL binding buffer containing 3.5 µL FITC Annexin v and 3.5 µL propidium iodide (PI) (Dojindo, Japan). Cells were incubated at room temperature for 15 min and analyzed by flow cytometry.

### Terminal deoxynucleotidyl transferase dUTP nick end labeling (TUNEL) assay

Apoptosis of gastric cancer cells was detected with One-Step TUNEL Apoptosis Kit (Green) (RiboAPO, Ribobio, China) according to the manufacturer's instructions. After TUNEL staining, the gastric cancer cells were stained using Hoechst and observed using a laser scanning confocal microscope (leica, Germany). The number of apoptotic cells is presented as a percentage of the total cells counted.

### Luciferase assay

The transcriptional activity was analyzed by the TopFlash and its mutant FopFlash (Addgene, USA) which were constructed by the TCF-responsive luciferase. TopFlash vector has 6 TCF binding sites, every 3 of which are in opposite directions. Negative control FopFlash had TCF binding site mutation. The relative activity of luciferase was determined by double luciferase reporter kit (Promega, USA). After transfection for 48 h, relative luciferase activity was determined and normalized to protein concentration. The results were the average of three independent experiments. We Used the PRL-TK report vector as the internal control vector. The statistical results came from three independent experiments.

### Immunofluorescence assay

The cells were fixed in 4% paraformaldehyde solution and 1% Triton solution was added to penetrate the cell membrane. Normal sheep serum ((DAKO, Germany) was then used to block the cells at room temperature for 30 minutes. After incubating with primary antibodies (Active-β-Catenin Antibody, Merck, Germany, Cat No: 05-665-25UG ,1:100) at 4°C for 12-16 hours, Primary antibody binding and nucleus were detected in the dark using fluorescence secondary antibody (1:100) and Hoechst staining kit, respectively. A laser scanning confocal microscope (Leica, Germany) was used to observe the cells. In each experiment, at least 200 cells were counted.

### Nuclear protein extraction assay

The assays were carried out according to the manufacturer's instructions for the Qproteome Cell Compartment Kit (Qiagen). 20 μg protein from each part was loaded into each well.10% SDS-PAGE and western blot were performed according to the above method.

### Co-immunoprecipitation (co-IP) assay

After MGC-803 and SGC-7901 cell lysates were centrifuged, the supernatant was added to 50% protein A/G agarose bead solution respectively according to the proportion of 100 µL of the bead solution to 1000 μL of the supernatant. Protein A/G agarose beards were discarded by transferring the supernatant to another tube after the non-specific binding proteins were removed from the beads. The TNFRSF11B and GSK3 β antibodies were used to pull down the proteins that interacted with TNFRSF11B and GSK3 β, respectively. And western blot was performed according to the above method. The IgG protein was used as positive control.

### Animal studies

Animal experiments were approved by the ethics committee of Peking University Cancer Hospital and Institute, and performed in accordance with the national and institutional guidelines. All animal experiments were conducted in compliance with animal protocols approved by Peking University Cancer Hospital and Institute, and were carried out at the Animal Center of Peking University Cancer Hospital and Institute. Five-week-old female BALB/c nude mice (BALB/c-nu) were purchased from Beijing Vital River Laboratory Animal Technology Co., Ltd.Their weights are 18±2 gram. Ten mice were randomly divided into two groups for the construction of Subcutaneous tumor formation model (five mice per group). The Scramble and shTNFRSF11B transfectants of MGC803 cells (4×10^5^ cells in 100 μL volume) were injected into both forelegs of BALB/c-nude mice. The tumors growth was monitored every 5 days and measured the width and length of the tumors with a caliper in two months. The vital signs of mice were observed also every 5 days after injection, no death was observed in the two groups. Two months later, the mice were euthanised with carbon dioxide, and the Subcutaneous tumor were removed. We do not dispose of the dead animal until it has been confirmed dead. We calculate the tumor volume by the formula V=0.5×L×W^2^ (L=length and W=width).

### Statistical analysis

Statistical analysis was performed with SPSS 24.0 (SPSS Inc., Chicago, IL, USA) and GraphPad prism 5.0. The data are expressed in the way of mean±SD error. Statistical tests were used by the student's *t*-test, one-way ANOVA and Chi-square test. *P*-value less than 0.05 was considered as a statistically significant difference for each statistical analysis.

## Results

### Bioinformatic analysis of TNFRSF11B expression in online databases

**Figure 1:**
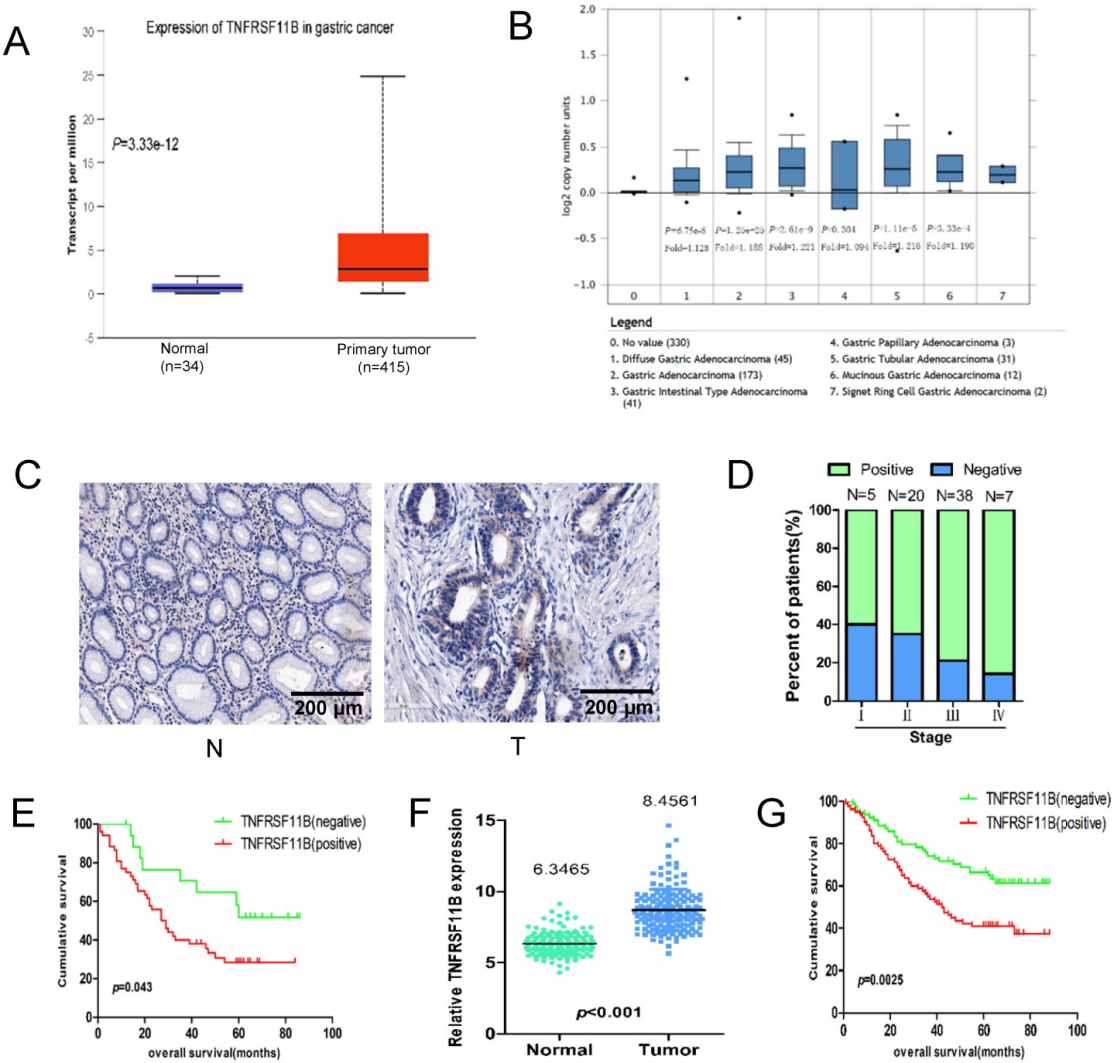
** The expression of TNFRSF11B in gastric cancer. (A)** The expression of TNFRSF11B was analyzed in the Cancer Genome Atlas (TCGA) project gastric cancer database, where a significant elevation in TNFRSF11B expression was observed in gastric adenocarcinoma tissues(n=415) compared to normal tissues(n=34). **(B)** The expression of TNFRSF11B was also analyzed in the Oncomine database where diffuse gastric adenocarcinoma (n=45), gastric adenocarcinoma(n=173), gastric intestinal type adenocarcinoma (n=41), gastric tubular adenocarcinoma (n=31) and mucinous gastric adenocarcinoma (n=12) were all found to be enhanced in comparison to normal tissues(n=330). **(C)** Expression of TNFRSF11B by immunohistochemical staining. Original magnification: 200×. N: normal stomach; T: gastric cancer **(D)** Association between TNFRSF11B expression status and TNM stage. **(E)** Kaplan-Meier survival curves of overall survival for 70 gastric cancer patients with TNFRSF11B-negative vs TNFRSF11B-positive GC tissue by IHC. **(F)** The expression of TNFRSF11B in primary GC (T) and corresponding surgical margin (N) tissues was examined by gene microarray analysis. **(G)** Kaplan-Meier survival curves of overall survival (OS) for 160 patients with TNFRSF11B-negative vs. TNFRSF11B-positive by gene microarray analysis.

The expression of TNFRSF11B was analyzed from the Cancer Genome Atlas (TCGA) project gastric cancer database and Oncomine database. Overall, we found that the expression of TNFRSF11B was higher in all of the gastric cancer tissues compared with normal tissues, suggesting that TNFRSF11B may be a crucial factor in gastric cancer (Fig. 1A and B).

### High expression of TNFRSF11B predicts a poor prognosis in GC patients

The expression of TNFRSF11B was measured by immunohistochemistry (IHC) in gastric cancer tissues. TNFRSF11B was rarely expressed in the normal gastric mucosa, but was highly expressed in the cytoplasm of gastric cancer tissues (Fig. 1C). The expression rate of TNFRSF11B in gastric cancer tissues was 74.3% (52/70). To elucidate the role of TNFRSF11B in the carcinogenesis of gastric cancer, we analyzed the relationship between TNFRSF11B expression and the clinicopathological characteristics of gastric cancer patients (Table 1).The frequency of TNFRSF11B expression increased remarkably with the progression of TNM stage (Fig.1D). However, there was no significant difference between TNFRSF11B expression and other clinicopathological parameters. The reasons may be the low number of cases.KM survival curve analysis showed that among the 70 patients, the 5-year OS rate of patients with TNFRSF11B positive expression was lower than that of patients with TNFRSF11B negative expression (*P*=0.043, Fig.1E). Multivariate Cox regression showed that there was not enough evidence to suggest the TNFRSF11B expression was an independent factor for the prognosis of gastric cancer patients (Table S1). It also may be due to a lack of cases.

The mRNA expression of TNFRSF11B was measured by gene microarray. TNFRSF11B mRNA expression in GC tissues was significantly higher than that in tissues from corresponding surgical margins (Fig. 1F, *P*<0.001).KM survival curve analysis showed that the 5-year OS rate of patients with high TNFRSF11B mRNA expression was lower than that of patients with low TNFRSF11B mRNA expression among the 160 patients (Fig.1G, *P*=0.0025). These results demonstrated that TNFRSF11B was highly expressed in the GC and associated with the patient poor outcome.

### Establishment of TNFRSF11B overexpression and knockdown in GC Cells

To further investigate the role of TNFRSF11B in GC, we first detected the expression level of TNFRSF11B in five GC cell lines. As shown in Fig. S1 A, we found that TNFRSF11B had low expression levels in HGC-27 and BGC-823 cells and had high expression levels in MGC-803 and SGC-7901 cells. We established stable cell lines transduced by the lentivirus carrying the TNFRSF11B gene. We overexpressed TNFRSF11B in HGC-27 and BGC-823 cell lines and knocked-down TNFRSF11B in MGC-803, SGC-7901 cell lines. Western blot analysis were used to verify overexpression and suppression of TNFRSF11B following transfection with appropriate plasmids. The protein expression of TNFRSF11B were significantly increased in HGC-27 and BGC-823 overexpression cells in comparison to control cells (Fig. S1 B and C).The TNFRSF11B protein expression were strongly inhibited in MGC-803 and SGC-7901 knockdown cells in comparison to control cells (Fig. S1 D and E).

### TNFRSF11B promotes cell growth *in vitro* and *in vivo*

**Figure 2:**
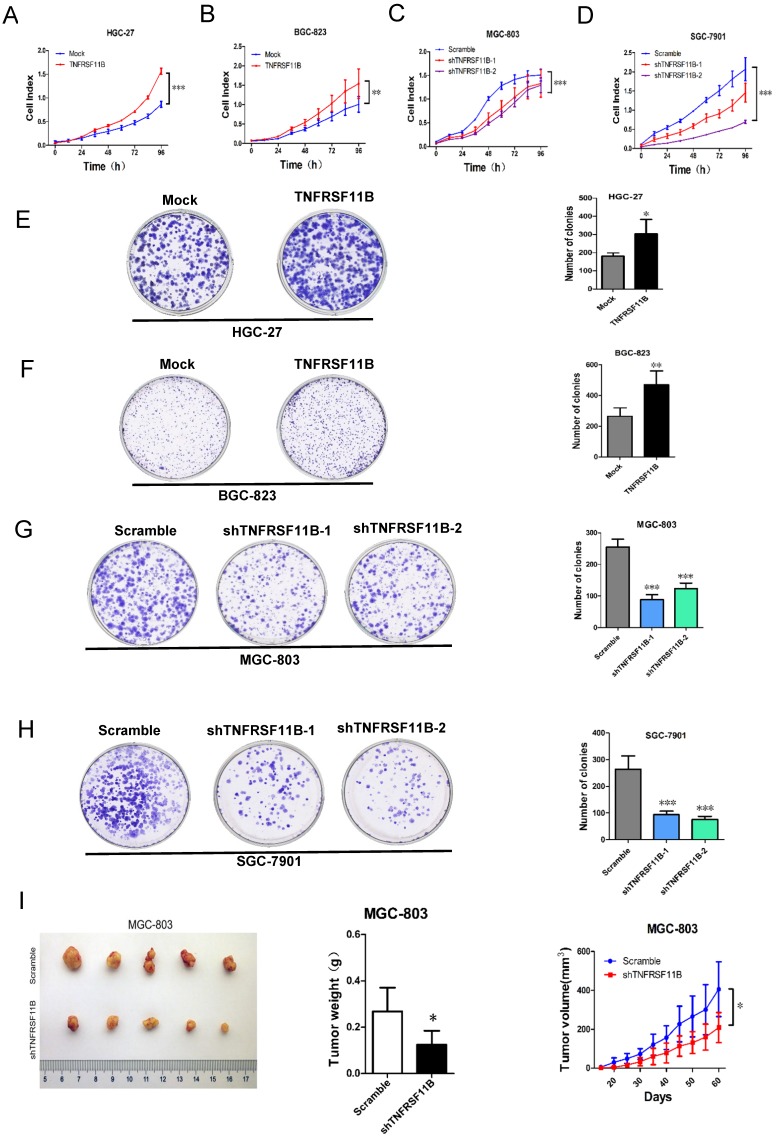
** TNFRSF11B promotes GC cell growth *in vitro* and *in vivo*. (A** and** B)** Proliferation activities were higher in HGC-27 and BGC-823 overexpression cells.** (C** and** D)** Proliferation activities **were** lower in MGC-803 and SGC-7901 knockdown cells. **(E** and** F)** Up-regulation of TNFRSF11B significantly increased cell clonogenicity in HGC-27 and BGC-823 cells. **(G and H)** Down-regulation of TNFRSF11B significantly reduced cell clonogenicity in MGC-803 and SGC-7901 cells. **(I)** Photograph showing tumor formation in nude mice injected with MGC803-Scramble and shTNFRSF11B, as well as tumor weights and tumor growth curve. The data are shown as mean ± SD. *p<0.05, **p<0.01, ***p<0.001

We examined the role of TNFRSF11B in proliferation by using the RTCA system. The results indicated that the proliferation activities were strongly promoted in HGC-27 and BGC-823 TNFRSF11B overexpression cells (Fig. 2 A and B). The proliferation activities were significantly inhibited in MGC-803 and SGC-7901 TNFRSF11B knockdown cells (Fig.2C and D). Moreover, we further investigated the TNFRSF11B in clonogenicity of GC cells by colony formation assay. Up-regulation of TNFRSF11B significantly increased cell clonogenicity in HGC-27 and BGC-823 cells (Fig. 2E and F). Down-regulation of TNFRSF11B significantly reduced cell clonogenicity in MGC-803 and SGC-7901 cells (Fig. 2G and H).To assess the tumorigenic ability of TNFRSF11B, MGC803 cells stably transfected with Scramble and shTNFRSF11B (shRNA 2#) was subcutaneously injected into the mice, respectively. There were five mice in the experimental group and five in the control group. They were killed after 2 months of observation. Knockdown of TNFRSF11B in MGC803 cells markedly reduced the tumor size (*P*=0.049) and weight of xenografts (*P*=0.014). And the growth of tumor was slower in the shTNFRSF11B group compared to the Scramble group (Fig. 2I). These in vitro and in vivo results support the tumor progression role of TNFRSF11B in GC.

### TNFRSF11B Promotes GC Cell Migration and Invasion

**Figure 3:**
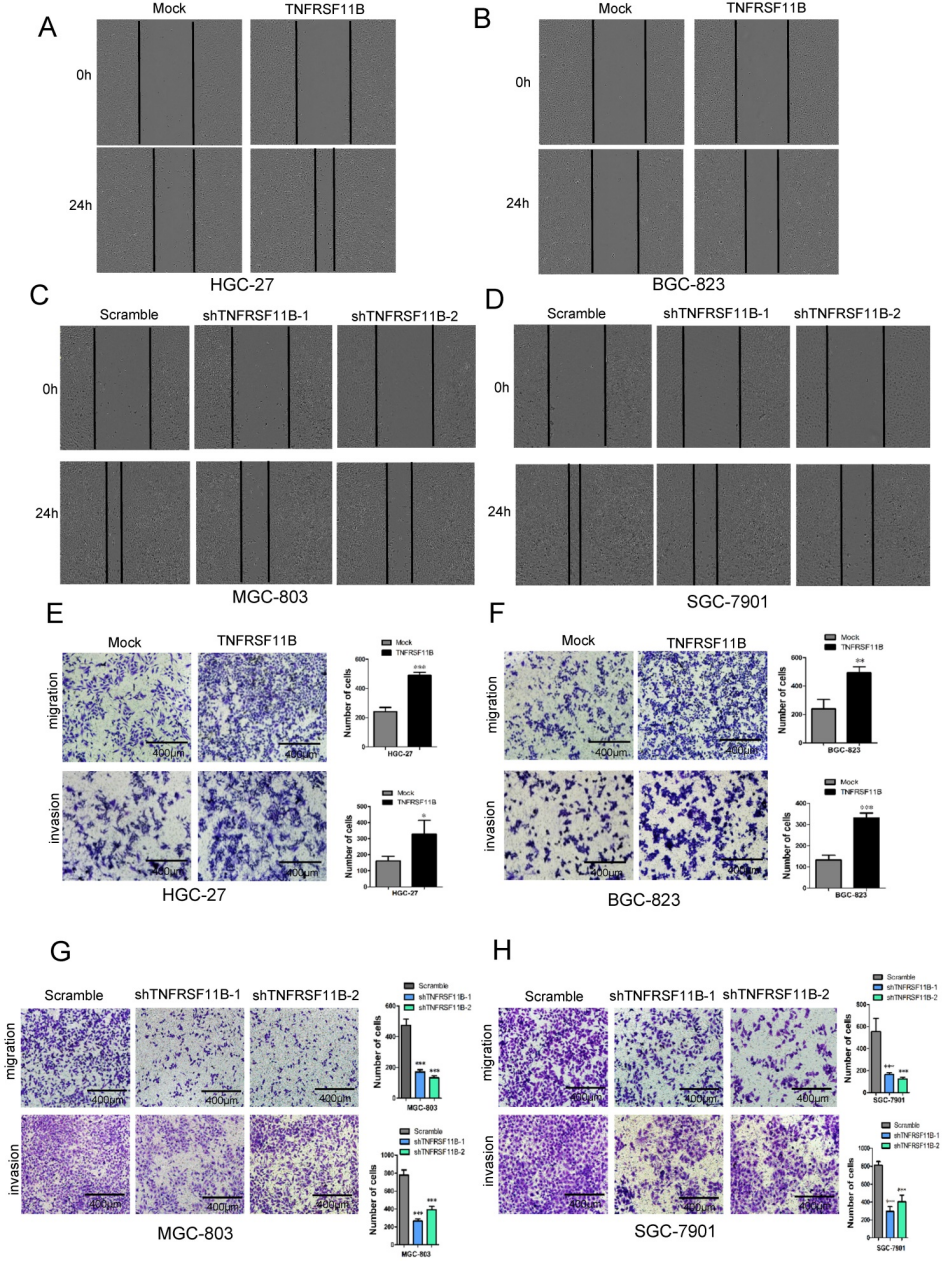
** TNFRSF11B promotes GC cell migration and invasion. (A** and** B)** In HGC-27 and BGC-823 overexpression cells, migration activities were significantly higher. **(C** and** D)** In MGC-803 and SGC-7901 cells, migration activities were significantly lower in TNFRSF11B knockdown cells. **(E** and** F)** TNFRSF11B overexpression increased migration and invasion of HGC-27 and BGC-823 cells in comparison to their respective control cells. **(G** and** H)** TNFRSF11B knockdown reduced migration and invasion ability in MGC-803 and SGC-7901 cells.*p**<**0.05, **p**<**0.01, ***p**<**0.001

We assessed the effect of TNFRSF11B in migration and invasion through the IncuCyte Zoom system and transwell chamber assay. We identified that the migration activities were significantly higher in HGC-27 and BGC-823 overexpression cells (Fig. 3A and B). And the migration activities were more lower in MGC-803 and SGC-7901 TNFRSF11B knockdown cells (Fig. 3Cand D). In transwell chamber assay, Up-regulation of TNFRSF11B significantly promoted cell migration and invasion in HGC-27 and BGC-823 cells (Fig. 3E and F). Down-regulation of TNFRSF11B markedly prevented cell migration and invasion in MGC-803 and SGC-7901 cells (Fig. 3G and H).

### Effects of TNFRSF11B on cell cycle and apoptosis in GC cells

**Figure 4:**
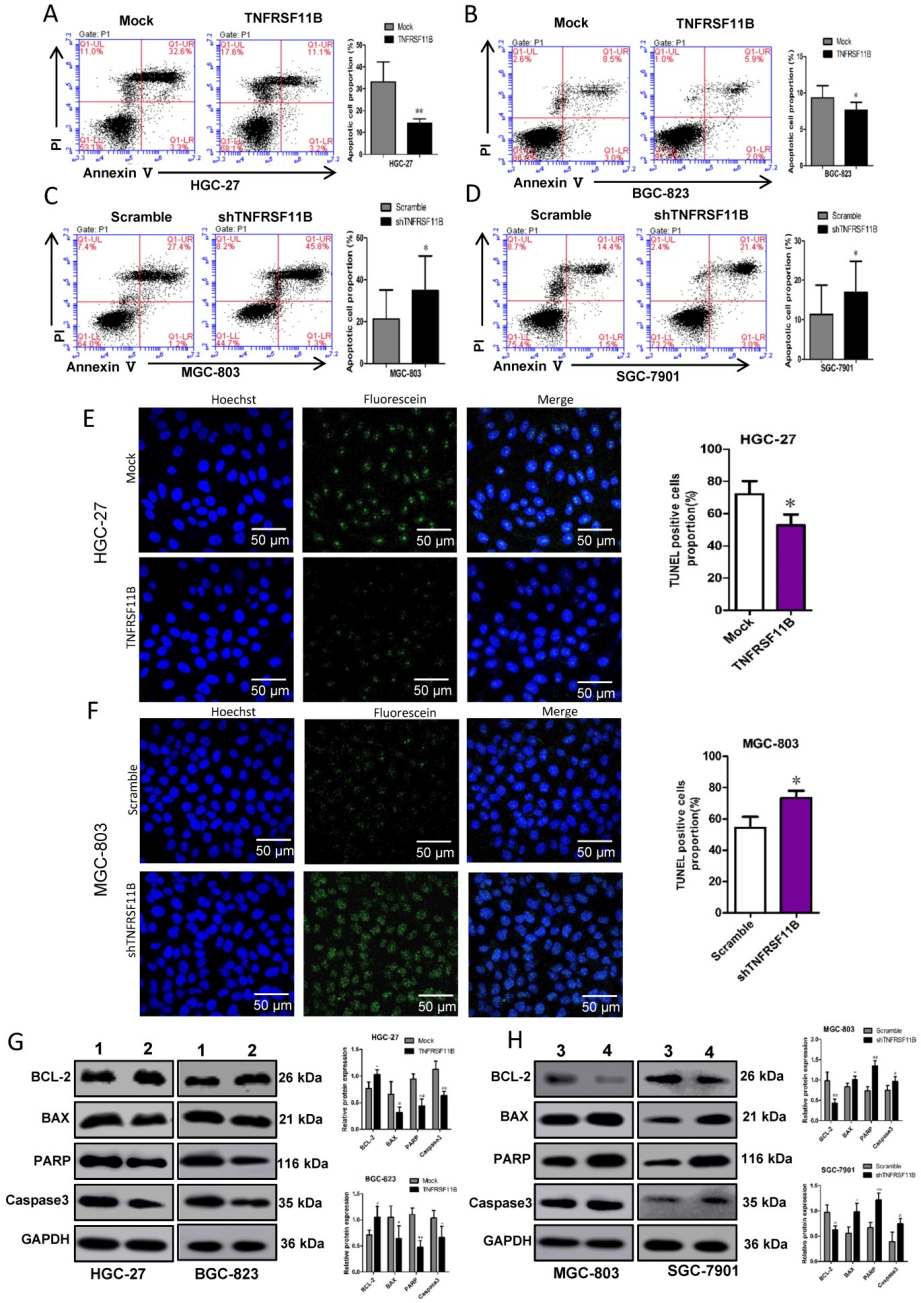
** Effects of TNFRSF11B on apoptosis in GC cells. (A and B)** TNFRSF11B overexpression cell groups showed decreased apoptosis rate in HGC-27 and BGC-823 cells. **(C and D)**TNFRSF11B knockdown cell groups showed increased apoptosis rate in MGC-803 and SGC7901 cells. **(E)** In TUNEL assay, the apoptotic cell rate in the HGC-27 TNFRSF11B overexpression group was significantly lower than the control group. **(F)** The apoptotic cell rate in the MGC-803 TNFRSF11B knockdown group was significantly higher than the control group. **(G)** The expression of BCL-2 was higher and the BAX, PARP and Caspase3 were lower in HGC-27 and BGC-823 TNFRSF11B overexpression cells than control cells. **(H)** The expression of BCL-2 was low and the BAX、PARP and Caspase3 were high in MGC-803 and SGC-7901 cells. 1: Mock, 2: TNFRSF11B, 3: Scramble, 4: shTNFRSF11B. *p<0.05, **p<0.01, ***p<0.001

We selected shRNA 2# cell lines with better knockout results for other related experiments (shTNFRSF11B is shTNFRSF11B-2).To certify whether TNFRSF11B manipulates cell growth by influencing DNA synthesis or apoptosis, we monitored cell cycle by fluorescence flow cytometry on a FACScan machine. We identified no significant differences in cell cycle progression in TNFRSF11B overexpression and knockdown cells versus control cells (Fig.S2 A-D). In contrast, apoptosis had significantly variation in TNFRSF11B overexpression and knockdown cells compared with control cells. Apoptosis was assessed by annexin V and PI staining in GC cells. TNFRSF11B- infected cell groups showed decreased apoptosis rate compared to their corresponding control ones in HGC-27 and BGC-823 cells (Figure. 4A and B) shTNFRSF11B-infected cell groups showed increased apoptosis rate compared to their corresponding control ones in MGC-803 and SGC7901 cells (Figure. 4C and D).

In TUNEL assay, the apoptotic cell rate in the HGC-27 and BGC-823 TNFRSF11B overexpression group was significantly lower than the control group (Fig. 4E; Fig. S3 A). The apoptotic cell rate in the MGC-803 and SGC-7901 TNFRSF11B knockdown group was significantly increased (Fig. 4F; Fig. S3 B).

Apoptosis associated protein, such as Caspase9, PARP, BCL-2, BAX and etc., were detected by western blot for further research. The results showed that the expression of BCL-2 was higher in HGC-27 and BGC- 823 TNFRSF11B overexpression cells than control cells. The expression of BAX, PARP and Caspase3 were lower in HGC-27 and BGC-823 TNFRSF11B overexpression cells(Fig. 4G). In contrast, there were the inverse results in MGC-803 and SGC-7901 TNFRSF11B knockdown cells (Fig. 4H). It could be because TNFRSF11B competed for the binding of TRAIL to death activated receptors, providing a mechanism to prevent apoptosis in the presence of TRAIL.

### TNFRSF11B activates Wnt/β-catenin signaling pathway

**Figure 5:**
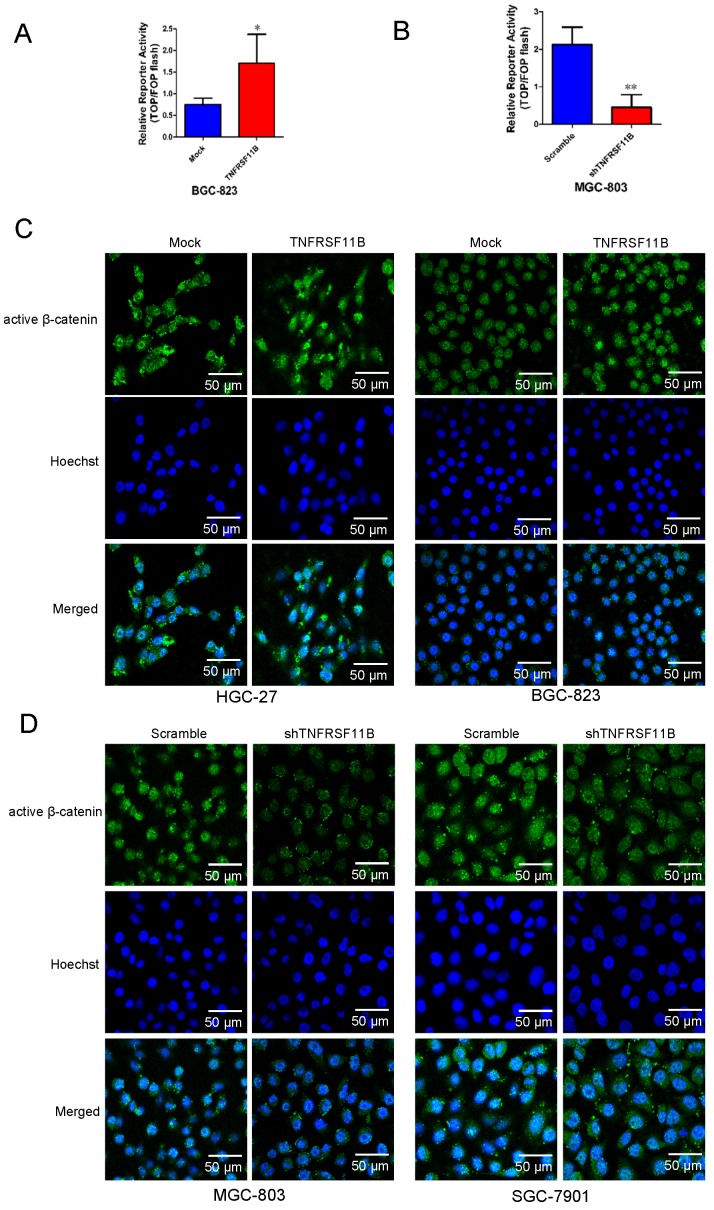
** TNFRSF11B affected β-catenin activity and the nuclear localization of β-catenin. (A)** Overexpression of TNFRSF11B significantly enhanced the transcriptional activity of β-catenin in BGC-823 cells by TOP-Flash reporter assay. **(B)** Knockdown of TNFRSF11B significantly suppressed the transcriptional activity of β-catenin in MGC-803 cells by TOP-Flash reporter assay. **(C and D)** The nuclear localization of active β-catenin in four GC cells was detected using immunofluorescent microscopy. The representative images are shown. The proportion of nuclear active β-catenin showed positively correlation with TNFRSF11B expression. *p<0.05, **p<0.01, ***p<0.001

**Figure 6:**
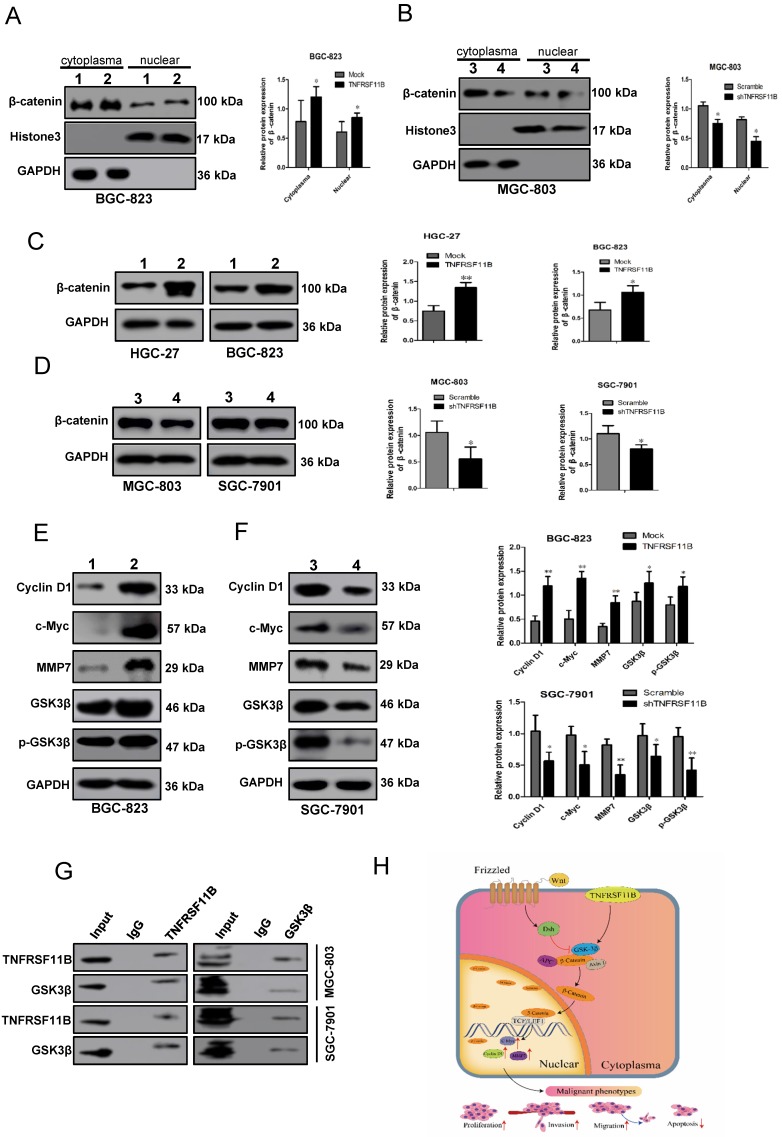
** TNFRSF11B increased β-catenin and related protein expression and hypothetic model for the mechanism of TNFRSF11B in gastric cancer. (A)** Overexpression of TNFRSF11B increased the protein level of cytoplasmic and nuclear β-catenin in BGC-823 cells.** (B)** Knockdown of TNFRSF11B decreased the protein level of cytoplasmic and nuclear β-catenin in MGC-803 cells. **(C)** Overexpression of TNFRSF11B increased β-catenin expression in HGC-27 and BGC-823 cells.** (D)** Knockdown of TNFRSF11B decreased β-catenin expression in MGC-803 and SGC-7901 cells. **(E)** Overexpression of TNFRSF11B increased the protein expression of CyclinD1, c-Myc, MMP-7, GSK3β, p-GSK3β in BGC-823 cells. **(F)** Knockdown of TNFRSF11B decreased the protein expression of CyclinD1, c-Myc, MMP-7, GSK3β, p-GSK3β in SGC-7901 cells. **(G)** The Co-immunoprecipitation analysis of MGC-803 and SGC-7901 GC cell lines showed mutual interaction between TNFRSF11B and GSK-3β. **(H)** Hypothetic model for the function and mechanism of TNFRSF11B in gastric cancer. 1:Mock, 2:TNFRSF11B, 3:Scramble, 4: shTNFRSF11B. *p<0.05, **p<0.01, ***p<0.001

Next, we researched the downstream effectors of TNFRSF11B in gastric cancer. Firstly, we used Top-flash and derived Fop-flash reporter assays to detect the transcriptional activity of β-catenin. Overexpression of TNFRSF11B significantly enhanced the transcriptional activity of β-catenin in BGC-823 cells (Fig. 5A), which was suppressed by knockdown of TNFRSF11B in MGC-803 cells (Fig. 5B). It suggested that TNFRSF11B may promote β-catenin activity. Secondly, the intracellular localization of active β-catenin was detected using fluorescent microscopy. The proportion of nuclear active β-catenin showed positively correlation with TNFRSF11B expression (Fig. 5C and D). The correlation between AXIN2, LGR5, LRP6 with TNFRSF11B expression in human GC samples was analyzed via TCGA database (GEPIA website: http://gepia.cancer-pku.cn/detail.php). We found that TNFRSF11B was significantly correlated with these genes (Fig. S4A). Moreover, these genes were analyzed in cell lines by qRT-PCR. Overexpression of TNFRSF11B increased the expression of AXIN2, LGR5 and LRP6 in BGC-823 cells (Fig. S4B). Knockdown of TNFRSF11B decreased the expression of AXIN2, LGR5 and LRP6 in MGC- 803 cells (Fig. S4C).The fractionation experiments proved the significant changes in β-catenin protein levels in both the nucleus and cytoplasm when TNFRSF11B was upregulated (Fig. 6A) or downregulated (Fig. 6B), indicating that TNFRSF11B may promote β-catenin by enhancing its protein expression. Direct detection of the β-catenin levels in total protein and its downstream targets provided additional evidence for this hypothesis. Overexpression of TNFRSF11B increased total β-catenin expression (Fig. 6C). Knockdown of TNFRSF11B decreased total β-catenin expression (Fig. 6D). Overexpression of TNFRSF11B increased the protein expression of CyclinD1, c-Myc, MMP-7, GSK3β, p-GSK3β in BGC-823 cells (Fig. 6E). Knockdown of TNFRSF11B decreased the protein expression of CyclinD1, c-Myc, MMP-7, GSK3β, p-GSK3β in SGC-7901 cells (Fig. 6F). These data further confirm that TNFRSF11B play significant effects on β-catenin protein expression.

The nuclear localization of β-catenin and protein expression is strictly regulated by GSK-3β.GSK-3β Phosphorylation at Ser9 results in decrease in β-catenin phosphorylation at Ser37/Thr41 and subsequent increase in β-catenin activity. Therefore, we detected the role of TNFRSF11B on GSK-3β phosphorylation. Overexpression of TNFRSF11B in GC cells led to an upregulation of GSK-3β phosphorylation at Ser9 (Fig. 6E). Nevertheless, knockdown of TNFRSF11B showed the opposite results (Fig. 6F). It was suggested that TNFRSF11B may promote the activity of β-catenin activity by upregulating GSK-3β phosphorylation at Ser9.The co-immunoprecipitation analysis of MGC-803 and SGC-7901 GC cell lines showed mutual interaction between TNFRSF11B and GSK-3β, which suggested that TNFRSF11B may regulate GSK-3β phosphorylation through protein interaction (Fig. 6 G).

Based on the above, these data revealed that TNFRSF11B promoted cell proliferation, migration and invasion in gastric cancer via Wnt/β-catenin pathway possibly and TNFRSF11B may regulate GSK-3β phosphorylation through protein interaction. The probable mechanism of TNFRSF11B inhibiting apoptosis is that TNFRSF11B competed for the binding of TRAIL to death activated receptors, providing a mechanism to prevent apoptosis in the presence of TRAIL (Fig. 6H).

## Discussion

TNFRSF11B expressed in various solid tumors and performs multiple effects in pathophysiological processes, which is closely related to the tumorigenesis and development of tumors. However, the knowledge on the expression patterns and biological function of TNFRSF11B remains limited in the progression of GC. In the present study, we found that the expression of TNFRSF11B was higher in gastric cancer tissues compared to normal tissues from the TCGA and Oncomine databases. In immunohistochemistry assay, we detected that the expression of TNFRSF11B was upregulated in gastric cancer. In order to clarify the role of TNFRSF11B in the pathogenesis of gastric cancer, we analyzed the relationship between the expression of TNFRSF11B and the clinicopathological characteristics of GC patients. TNFRSF11B obviously correlated with TNM stage. However, there was no significant difference between TNFRSF11B expression and other clinicopathological parameters. The reason might be small number of GC samples and we need larger cases for further study. The results of immunohistochemistry and gene microarray analysis indicated that TNFRSF11B was correlated with poor prognosis. These data support similar trends that have been reported in a wide range of solid tumors, including breast, pancreas, colorectum 13, 25, 26.

To further certify the effects of TNFRSF11B in gastric carcinogenesis and progression, the roles of upregulating and downregulating TNFRSF11B on cell function were assessed. In our research, the results showed that the TNFRSF11B promoted cell proliferation, migration, invasion and unlimited growth *in vitro* and tumorigenic ability *in vitro* and *in vivo*.

A number of studies have suggested that TNFRSF11B was also considered as a survival factor for tumor cells inhibiting tumor cell apoptosis 9. *In vitro* studies suggested that TNFRSF11B exerted tumor-promoting effects by binding to TRAIL 27-29, thereby preventing induction of apoptosis 30. Our research showed that apoptosis had significant variation in TNFRSF11B overexpression and knockdown cells compared with control cells in flow cytometric analysis, TUNEL assay and western blot. The results demonstrated that TNFRSF11B inhibited apoptosis in GC cells. It maybe indicate that TNFRSF11B mediates resistance to TRAIL-induced apoptosis, thereby preventing induction of apoptosis. It requires further research for us. However, we identified that TNFRSF11B had no significant effect on cell cycle distribution in GC cells.

The Wnt/β -catenin signaling pathway is known as the primary driver of cancer development in gastric cancer 31, 32. TNFRSF11B may offer a survival protection on TNFRSF11B-producing tumor cells and contribute to tumorigenesis and the survival of cancer cell via driving TNFRSF11B expression by the Wnt/ β-catenin pathway 13, 24. Our study confirmed that TNFRSF11B increased β-catenin and related protein expression. And the proportion of nuclear active β-catenin showed positively correlation with TNFRSF11B expression. TNFRSF11B can upregulate GSK-3β phosphorylation. However, it is not clear how TNFRSF11B promotes GSK-3β phosphorylation. It has been reported that the phosphorylation of GSK-3β at Ser9 is regulated by several kinases, including protein kinases A, B and C 33, 34. One possibility is that TNFRSF11B directly or indirectly activates the activity of these kinases. Another one is that TNFRSF11B competes with these kinases for binding sites in GSK-3β. This will physically isolate these kinases from GSK-3β. Our research suggested that TNFRSF11B may regulate GSK-3β phosphorylation through protein interaction. So we speculated that TNFRSF11B may compete with these kinases for binding sites by binding with GSK-3β.

More work is needed to demonstrate the exact molecular mechanism of TNFRSF11B-mediated GSK-3β phosphorylation regulation. The transcription complex of β-catenin/TCF regulates the expression of a variety of downstream target genes, including crucial oncogenes such as MMP-7, Cyclin D1 and c-Myc 35, 36. It is of great significance to clarify that the β-catenin downstream targets are involved in mediating its carcinogenic effect in gastric cancer. These studies will further contribute to the analysis of the molecular mechanisms of β-catenin in gastric cancer.

Our research systematically assessed the effects of TNFRSF11B in gastric cancer and found the novel regulation of the GSK3β/β-catenin pathway through TNFRSF11B. These researches suggest that TNFRSF11B may have potential as a biomarker and clinical target in gastric cancer, warranting further study into the biological role of TNFRSF11B in gastric cancer. Inhibition of TNFRSF11B expression may provide a novel treatment for gastric tumor patients with overexpression of TNFRSF11B.

## Supplementary Material

Supplementary figures and tables.

## Figures and Tables

**Table 1:** Relationship between TNFRSF11B expression and clinicopathological features in patients with gastric cancer

Clinicopathological Features	TNFRSF11B expression		χ^2^	*P*
	Negative (%)	Positive (%)		
**Gender**			1.264	0.261
Male	11 (22.0)	39 (78.0)		
Female	7 (35.0)	13 (65.0)		
**Age, year**			0.286	0.593
≤60	11(28.2)	28 (71.8)		
>60	7(22.6)	24 (77.4)		
**Tumor location**			0.005	0.944
Cardiac	5 (26.3)	14 (73.7)		
Non cardiac	13(25.5)	38 (74.5)		
**Tumor size**			0.654	0.419
≤4cm	12 (29.3)	29 (70.7)		
>4cm	6 (20.7)	23(79.3)		
**Differentiation**			0.974	0.807
Low	8 (22.9)	27 (77.1)		
Medium-low	7 (29.2)	17 (70.8)		
Medium	2 (22.2)	7(77.8)		
High	1(50)	1(50)		
**Vascular invasion**			0.321	0.571
Absent	9 (29.0)	22(71.0)		
Present	9(23.1)	30 (76.9)		
**Depth of invasion**			0.033	0.855
T1-2	2(28.6)	5(71.4)		
T3-4	16 (25.4)	47(74.6)		
**Lymph node**			2.372	0.499
N0	4(30.8)	9(69.2)		
N1	4(40.0)	6(60.0)		
N2	5(27.8)	13(72.2)		
N3	5(17.2)	24(82.8)		
**Distant metastasis**			0.532	0.466
M0	17 (27.0)	46 (73.0)		
M1	1 (14.3)	6 (85.7)		
**TNM stage**			2.348	0.503
I	2 (40.0)	3(60.0)		
II	7 (35.0)	13 (65.0)		
III	8(21.1)	30 (78.9)		
IV	1(14.3)	6(85.7)		

Note: *P<0.05. Abbreviations: TNFRSF11B: Tumor necrosis factor receptor superfamily member 11B

## Abbreviations

TNFRSF11B: Tumor necrosis factor receptor superfamily member 11B
GC: gastric cancer
OPG: Osteoprotegerin
TRAIL: tumor necrosis factor related apoptosis inducing ligand
NF-kB: nuclear factor-kappa B
TCGA: the Cancer Genome Atlas
FBS: Fetal bovine serum
AJCC: American Joint Committee on Cancer
OS: Overall survival
TUNEL: Terminal deoxynucleotidyl transferase dUTP nick end labeling
co-IP: Co-immunoprecipitation

## References

[1] Bray F, Ferlay J, Soerjomataram I, Siegel RL, Torre LA, Jemal A (2018). Global cancer statistics 2018: GLOBOCAN estimates of incidence and mortality worldwide for 36 cancers in 185 countries. CA Cancer J Clin.

[2] Ma YT, Xing XF, Dong B, Cheng XJ, Guo T, Du H (2018). Higher autocrine motility factor/glucose-6-phosphate isomerase expression is associated with tumorigenesis and poorer prognosis in gastric cancer. Cancer Manag Res.

[3] Lauren P (1965). The Two Histological Main Types of Gastric Carcinoma: Diffuse and So-Called Intestinal-Type Carcinoma. An Attempt at a Histo-Clinical Classification. Acta Pathol Microbiol Scand.

[4] Patru CL, Surlin V, Georgescu I, Patru E (2013). Current issues in gastric cancer epidemiology. Rev Med Chir Soc Med Nat Iasi.

[5] Liang XJ, Chen C, Zhao Y, Wang PC (2010). Circumventing tumor resistance to chemotherapy by nanotechnology. Methods Mol Biol.

[6] Goswami S, Sharma-Walia N (2016). Osteoprotegerin rich tumor microenvironment: implications in breast cancer. Oncotarget.

[7] Maginn EN, Browne PV, Hayden P, Vandenberghe E, MacDonagh B, Evans P (2011). PBOX-15, a novel microtubule targeting agent, induces apoptosis, upregulates death receptors, and potentiates TRAIL-mediated apoptosis in multiple myeloma cells. Br J Cancer.

[8] Weichhaus M, Chung ST, Connelly L (2015). Osteoprotegerin in breast cancer: beyond bone remodeling. Mol Cancer.

[9] Holen I, Shipman CM (2006). Role of osteoprotegerin (OPG) in cancer. Clin Sci (Lond).

[10] Schoppet M, Preissner KT, Hofbauer LC (2002). RANK ligand and osteoprotegerin: paracrine regulators of bone metabolism and vascular function. Arterioscler Thromb Vasc Biol.

[11] Yu Z, Sanders AJ, Owen S, Cheng S, Yang X, Jiang WG (2017). Expression of Osteoprotegrin Is Enhanced in Lung Cancer Tissues and Promotes Aggressive Cellular Traits in H3122 Lung Cancer Cells. Anticancer Res.

[12] Wang S, El-Deiry WS (2003). TRAIL and apoptosis induction by TNF-family death receptors. Oncogene.

[13] De Toni EN, Thieme SE, Herbst A, Behrens A, Stieber P, Jung A (2008). OPG is regulated by beta-catenin and mediates resistance to TRAIL-induced apoptosis in colon cancer. Clin Cancer Res.

[14] Anselmino N, Starbuck M, Labanca E, Cotignola J, Navone N, Gueron G (2020). Heme Oxygenase-1 Is a Pivotal Modulator of Bone Turnover and Remodeling: Molecular Implications for Prostate Cancer Bone Metastasis. Antioxid Redox Signal.

[15] Han Z, Zhan R, Chen S, Deng J, Shi J, Wang W (2020). miR-181b/Oncostatin m axis inhibits prostate cancer bone metastasis via modulating osteoclast differentiation. J Cell Biochem.

[16] Cheng K, Shi J, Liu Z, Jia Y, Qin Q, Zhang H (2020). A panel of five plasma proteins for the early diagnosis of hepatitis B virus-related hepatocellular carcinoma in individuals at risk. EBioMedicine.

[17] Kang J, Choi YJ, Seo BY, Jo U, Park SI, Kim YH (2019). A Selective FGFR inhibitor AZD4547 suppresses RANKL/M-CSF/OPG-dependent ostoclastogenesis and breast cancer growth in the metastatic bone microenvironment. Sci Rep.

[18] Aversa J, Song M, Shimazu T, Inoue M, Charvat H, Yamaji T (2019). Prediagnostic circulating inflammation biomarkers and esophageal squamous cell carcinoma: A case-cohort study in Japan. Int J Cancer.

[19] Deligiorgi MV, Panayiotidis MI, Griniatsos J, Trafalis DT (2019). Harnessing the versatile role of OPG in bone oncology: counterbalancing RANKL and TRAIL signaling and beyond. Clin Exp Metastasis.

[20] Yeh IJ, Chen SC, Yen MC, Wu YH, Hung CH, Kuo PL (2019). 6-Shogaol Suppresses 2-Amino-1-Methyl-6-Phenylimidazo [4,5-b] Pyridine (PhIP)-Induced Human 786-O Renal Cell Carcinoma Osteoclastogenic Activity and Metastatic Potential. Nutrients.

[21] Wieser V, Sprung S, Tsibulak I, Haybaeck J, Hackl H, Fiegl H (2019). Clinical Impact of RANK Signalling in Ovarian Cancer. Cancers (Basel).

[22] Ito R, Nakayama H, Yoshida K, Kuraoka K, Motoshita J, Oda N (2003). Expression of osteoprotegerin correlates with aggressiveness and poor prognosis of gastric carcinoma. Virchows Arch.

[23] D'Amico L, Satolli MA, Mecca C, Castiglione A, Ceccarelli M, D'Amelio P (2013). Bone metastases in gastric cancer follow a RANKL-independent mechanism. Oncol Rep.

[24] Wang X, Liu Y, Shao D, Qian Z, Dong Z, Sun Y (2016). Recurrent amplification of MYC and TNFRSF11B in 8q24 is associated with poor survival in patients with gastric cancer. Gastric Cancer.

[25] Shi W, Qiu W, Wang W, Zhou X, Zhong X, Tian G (2014). Osteoprotegerin is up-regulated in pancreatic cancers and correlates with cancer-associated new-onset diabetes. Biosci Trends.

[26] Chung ST, Geerts D, Roseman K, Renaud A, Connelly L (2017). Osteoprotegerin mediates tumor-promoting effects of Interleukin-1beta in breast cancer cells. Mol Cancer.

[27] Lane D, Matte I, Rancourt C, Piche A (2012). Osteoprotegerin (OPG) protects ovarian cancer cells from TRAIL-induced apoptosis but does not contribute to malignant ascites-mediated attenuation of TRAIL-induced apoptosis. J Ovarian Res.

[28] Renema N, Navet B, Heymann MF, Lezot F, Heymann D (2016). RANK-RANKL signalling in cancer. Biosci Rep.

[29] Sisay M, Mengistu G, Edessa D (2017). The RANK/RANKL/OPG system in tumorigenesis and metastasis of cancer stem cell: potential targets for anticancer therapy. Onco Targets Ther.

[30] Malyankar UM, Scatena M, Suchland KL, Yun TJ, Clark EA, Giachelli CM (2000). Osteoprotegerin is an alpha vbeta 3-induced, NF-kappa B-dependent survival factor for endothelial cells. J Biol Chem.

[31] Wang JB, Wang ZW, Li Y, Huang CQ, Zheng CH, Li P (2017). CDK5RAP3 acts as a tumor suppressor in gastric cancer through inhibition of beta-catenin signaling. Cancer Lett.

[32] Ganesan K, Ivanova T, Wu Y, Rajasegaran V, Wu J, Lee MH (2008). Inhibition of gastric cancer invasion and metastasis by PLA2G2A, a novel beta-catenin/TCF target gene. Cancer Res.

[33] Cross DA, Alessi DR, Cohen P, Andjelkovich M, Hemmings BA (1995). Inhibition of glycogen synthase kinase-3 by insulin mediated by protein kinase B. Nature.

[34] Sutherland C, Cohen P (1994). The alpha-isoform of glycogen synthase kinase-3 from rabbit skeletal muscle is inactivated by p70 S6 kinase or MAP kinase-activated protein kinase-1 in vitro. FEBS Lett.

[35] He TC, Sparks AB, Rago C, Hermeking H, Zawel L, da Costa LT (1998). Identification of c-MYC as a target of the APC pathway. Science.

[36] Shtutman M, Zhurinsky J, Simcha I, Albanese C, D'Amico M, Pestell R (1999). The cyclin D1 gene is a target of the beta-catenin/LEF-1 pathway. Proc Natl Acad Sci U S A.

