# Supplementary material for: TNFRSF11B activates Wnt/β-catenin signaling and promotes gastric cancer progression
Source: Int J Biol Sci. 2020 Apr 25;16(11):1956–71. doi: 10.7150/ijbs.43630 (PMC7211174; doi:10.7150/ijbs.43630)
Supplement: Supplementary file 1 — Supplementary figures and tables. [file ijbsv16p1956s1.pdf]

.

Supplementary Figure Legends

Figure S1. The expression of TNFRSF11B in GC cells. **(A)** The mRNA expression of TNFRSF11B in GC cell lines**. (B** and **C)** The protein expression of TNFRSF11B was enhanced in HGC-27 and BGC-823 TNFRSF11B overexpression groups in comparison to the Mock groups. **(D** and **E)**The protein expression of TNFRSF11B was decreased in MGC-803 and SGC-7901 TNFRSF11B knockdown groups.

Figure S2. Gastric cancer cell cycle analysis by flow cytometry. **(A** and **B)** In cell cycle progression, there is no significant differences in HGC-27 and BGC-823 TNFRSF11B overexpression cells versus control cells (P>0.05). **(C** and **D)** There is no significant differences in MGC-803 and SGC7901 knockdown cells versus control cells (P>0.05). .*p<0.05, **p<0.01, ***p<0.001, ns: no significance

Figure S3. TUNEL assay for gastric cancer cells. **(A)** The apoptotic cell rate in the BGC-823 TNFRSF11B overexpression group was significantly lower than the control group. **(B)**The apoptotic cell rate in the SGC-7901 TNFRSF11B knockdown group was significantly higher than the control group. .*p<0.05, **p<0.01, ***p<0.001

Figure S4. The correlation between AXIN2, LGR5, LRP6 with TNFRSF11B expression in human GC.
(A) TNFRSF11B was significantly correlated with AXIN2, LGR5 and LRP6 in TCGA database.
(B) Overexpression of TNFRSF11B increased the expression of AXIN2, LGR5 and LRP6 in BGC-823 cells. **(C)** Knockdown of TNFRSF11B decreased the expression of AXIN2, LGR5 and LRP6 in MGC-803 cells.

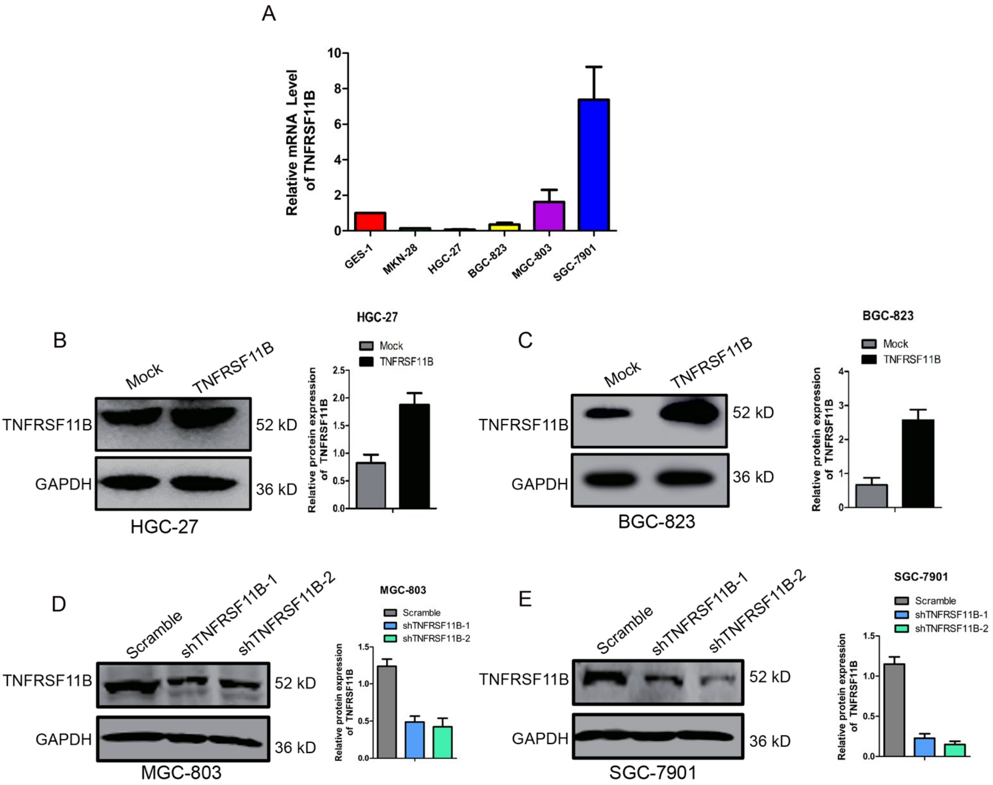
A
Relative mRNA Level of TNFRSF11B
GES-1
MKN-28
HGC-27
BGC-823
MGC-803
SGC-7901
B
Mock
TNFRSF11B
TNFRSF11B
GAPDH
52 kD
36 kD
HGC-27
HGC-27
Mock
TNFRSF11B
Relative protein expression of TNFRSF11B
C
Mock
TNFRSF11B
TNFRSF11B
GAPDH
52 kD
36 kD
BGC-823
BGC-823
Mock
TNFRSF11B
Relative protein expression of TNFRSF11B
D
Scramble
shTNFRSF11B-1
shTNFRSF11B-2
TNFRSF11B
GAPDH
52 kD
36 kD
MGC-803
MGC-803
Scramble
shTNFRSF11B-1
shTNFRSF11B-2
Relative protein expression of TNFRSF11B
E
Scramble
shTNFRSF11B-1
shTNFRSF11B-2
TNFRSF11B
GAPDH
52 kD
36 kD
SGC-7901
SGC-7901
Scramble
shTNFRSF11B-1
shTNFRSF11B-2
Relative protein expression of TNFRSF11B

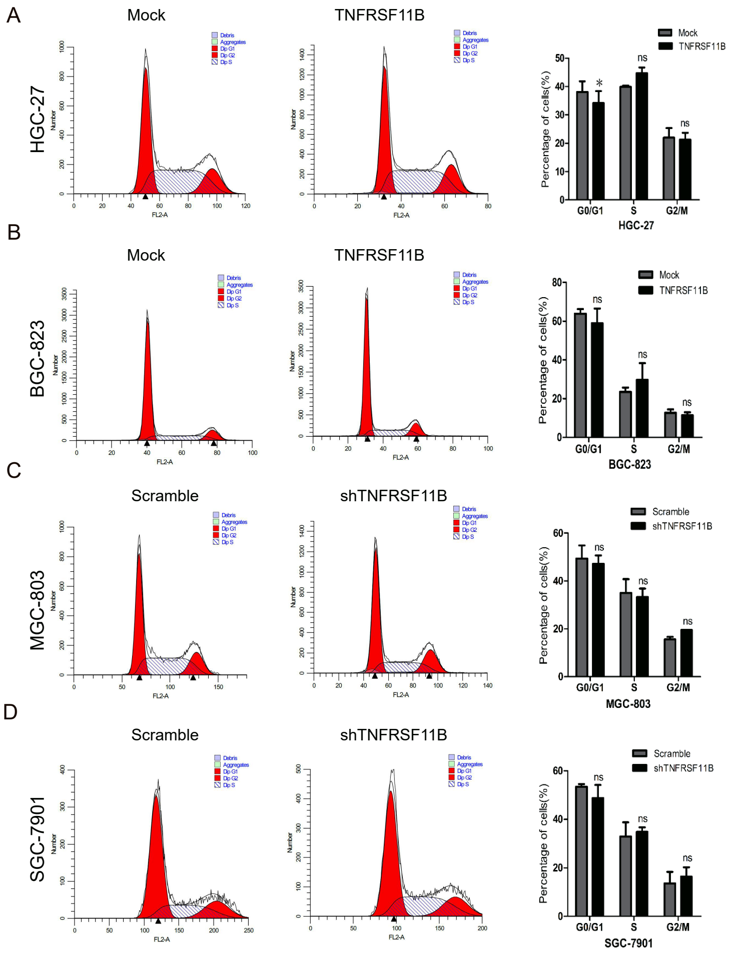

A
Mock
TNFRSF11B
HGC-27
Debris
Aggregates
Dip G1
Dip G2
Dip S
Number
FL2-A
Mock
TNFRSF11B
Percentage of cells(%)
G0/G1
S
G2/M
HGC-27
*
ns
B
Mock
TNFRSF11B
BGC-823
BGC-823
C
Scramble
shTNFRSF11B
MGC-803
MGC-803
D
Scramble
shTNFRSF11B
SGC-7901
SGC-7901

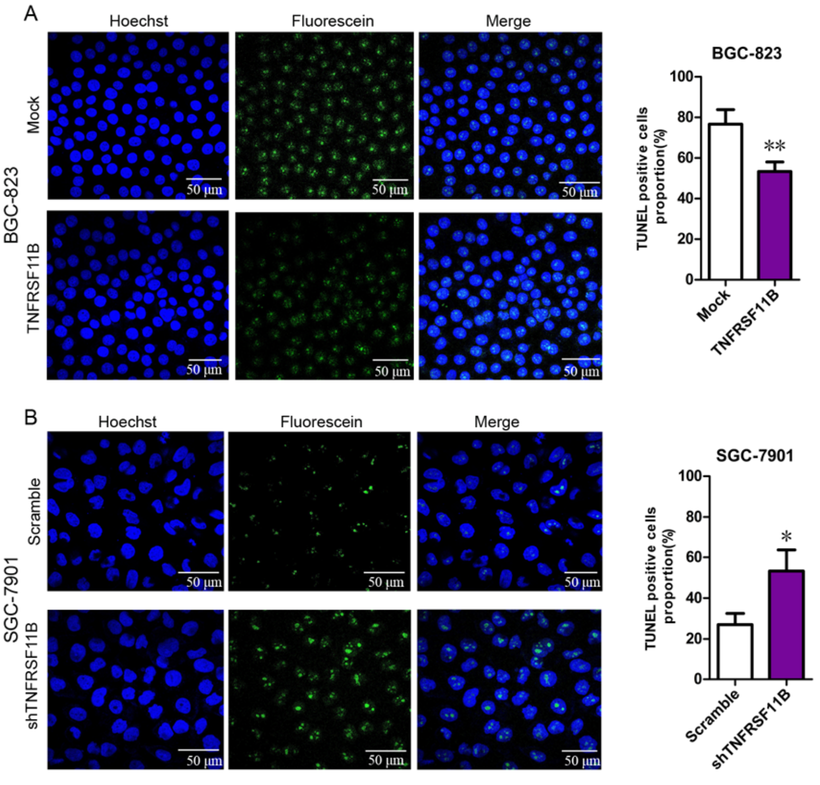

A
Hoechst
Fluorescein
Merge
BGC-823
Mock
TNFRSF11B
50 μm
BGC-823
TUNEL positive cells proportion(%)
100
80
60
40
20
0
**
Mock
TNFRSF11B
B
Hoechst
Fluorescein
Merge
SGC-7901
Scramble
shTNFRSF11B
50 μm
SGC-7901
TUNEL positive cells proportion(%)
100
80
60
40
20
0
*
Scramble
shTNFRSF11B

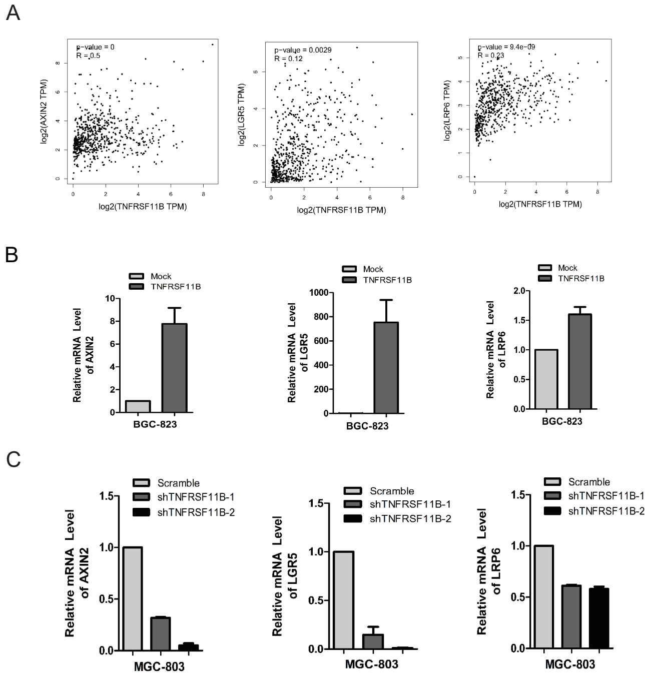
A
p-value = 0
R = 0.5
log2(AXIN2 TPM)
log2(TNFRSF11B TPM)
p-value = 0.0029
R = 0.12
log2(LGR5 TPM)
log2(TNFRSF11B TPM)
p-value = 9.4e-09
R = 0.23
log2(LRP6 TPM)
log2(TNFRSF11B TPM)
B
Mock
TNFRSF11B
Relative mRNA Level of AXIN2
BGC-823
Relative mRNA Level of LGR5
BGC-823
Relative mRNA Level of LRP6
BGC-823
C
Scramble
shTNFRSF11B-1
shTNFRSF11B-2
Relative mRNA Level of AXIN2
MGC-803
Relative mRNA Level of LGR5
MGC-803
Relative mRNA Level of LRP6
MGC-803

Table S1 Predictors of mortality using the univariate and multivariate Cox proportional hazard model among participants (n=70)

Covariates	Univariate analysis			Multivariate analysis		
	HR	95%CI	*P*	HR	95%CI	*P*
**Gender**						
Male	Reference					
Female	1.052	0.541-2.044	0.882			
**Age, years**						
≤60	Reference					
>60	1.887	1.041-3.422	0.037*	1.601	0.754-3.397	0.220
**Tumor location**						
Cardiac	Reference					
Non cardiac	0.597	0.318-1.121	0.109			
**Tumor size**						
≤4cm	Reference					
>4cm	2.111	1.162-3.838	0.014*	1.646	0.835-3.244	0.150
**Differentiation**						
Low	Reference					
Medium-low	0.916	0.475-1.767	0.794			
Medium	0.924	0.349-2.448	0.874			
High	1.519	0.357-6.471	0.572			
**Vascular invasion**						
Absent	Reference					
Present	2.186	1.170-4.086	0.014*	1.199	0.548-2.622	0.650
**Depth of invasion**						
T1-2	Reference					
T3-4	26.543	0.748-941.847	0.072			
**Lymph node**						
N0	Reference					
N1	2.046	0.342-12.252	0.433			
N2	7.942	1.781-35.416	0.007*	7.086	1.442-34.823	0.016*
N3	11.727	2.766-49.720	0.001*	12.027	2.498-57.920	0.002*
**Distant metastasis**						
M0	Reference					
M1	3.174	1.393-7.232	0.006*	1.074	0.399-2.896	0.887
**TNFRSF11B expression**						
Negative	Reference					
Positive	2.160	1.000-4.664	0.050			

Note: *P<0.05

Abbreviations: TNFRSF11B，Osteoprotegerin.
